# The costs of providing antiretroviral therapy services to HIV-infected individuals presenting with advanced HIV disease at public health centres in Dar es Salaam, Tanzania: Findings from a randomised trial evaluating different health care strategies

**DOI:** 10.1371/journal.pone.0171917

**Published:** 2017-02-24

**Authors:** Godfather Dickson Kimaro, Sayoki Mfinanga, Victoria Simms, Sokoine Kivuyo, Christian Bottomley, Neil Hawkins, Thomas S. Harrison, Shabbar Jaffar, Lorna Guinness, on behalf of the REMSTART trial team

**Affiliations:** 1 Muhimbili Medical Research Centre, National Institute for Medical Research, Dar es Salaam, Tanzania; 2 Department of Infectious Disease Epidemiology, London School of Hygiene and Tropical Medicine, London, United Kingdom; 3 Department of Global Health and Development, London School of Hygiene and Tropical Medicine, London, United Kingdom; 4 Institute for Infection and Immunity, St Georges University of London, London, United Kingdom; 5 Department of International Public Health, Liverpool School of Tropical Medicine, Liverpool, United Kingdom; Azienda Ospedaliera Universitaria di Perugia, ITALY

## Abstract

**Background:**

Understanding the costs associated with health care delivery strategies is essential for planning. There are few data on health service resources used by patients and their associated costs within antiretroviral (ART) programmes in Africa.

**Material and methods:**

The study was nested within a large trial, which evaluated screening for cryptococcal meningitis and tuberculosis and a short initial period of home-based adherence support for patients initiating ART with advanced HIV disease in Tanzania and Zambia. The economic evaluation was done in Tanzania alone. We estimated costs of providing routine ART services from the health service provider's perspective using a micro-costing approach. Incremental costs for the different novel components of service delivery were also estimated. All costs were converted into US dollars (US$) and based on 2012 prices.

**Results:**

Of 870 individuals enrolled in Tanzania, 434 were enrolled in the intervention arm and 436 in the standard care/control arm. Overall, the median (IQR) age and CD4 cell count at enrolment were 38 [31, 44] years and 52 [20, 89] cells/mm3, respectively. The mean per patient costs over the first three months and over a one year period of follow up following ART initiation in the standard care arm were US$ 107 (95%CI 101–112) and US$ 265 (95%CI 254–275) respectively. ART drugs, clinic visits and hospital admission constituted 50%, 19%, and 19% of the total cost per patient year, while diagnostic tests and non-ART drugs (co-trimoxazole) accounted for 10% and 2% of total per patient year costs. The incremental costs of the intervention to the health service over the first three months was US$ 59 (p<0.001; 95%CI 52–67) and over a one year period was US$ 67(p<0.001; 95%CI 50–83). This is equivalent to an increase of 55% (95%CI 51%–59%) in the mean cost of care over the first three months, and 25% (95%CI 20%–30%) increase over one year of follow up.

## Background

Over 12 million HIV-infected people now have access to antiretroviral therapy (ART) in Africa [1] where there are severe constraints on health care resources, in particular, a severe shortage of health care workers. Understanding the costs associated with different approaches to health care delivery is essential to inform policy, and especially important in resource-limited settings [2]. Despite this, there are surprisingly few published studies describing the costs of ART programmes in low income countries in Africa [3].

Costing studies use either the “top-down” or “bottom-up” approach or a combination of these [4,5]. The former estimates costs by dividing the past expenditure by the number of services provided during the period that expenditure was incurred. These analyses are crude and it is not possible to assess how patient characteristics influence costs and or the extent to which costs vary between settings. The “bottom up” approach, also referred to as the ingredient-based approach, quantifies the inputs used to provide the service outputs. Micro-costing is a form of “bottom up” approach and is essential for evaluating new interventions [4,6,7] as it allows for the statistical analysis of the key cost drivers at the individual level. Since 2003, just four published HIV costing studies from low income sub-Saharan Africa have used an individual level micro-costing approach, but all were retrospective [8–11] and their findings are now outdated [12–16].

## Materials and methods

In this study, we estimated the costs of ART delivery in the primary care setting in Tanzania using a micro-costing approach. Our study was nested within a trial, which aimed to reduce HIV-mortality among patients presenting in the very advanced stages of HIV-infection (REMSTART) [17]. Half of the participants received routine standard care. The other half received routine standard care combined with additional screening and adherence support. The participants were managed by health care staff according to national guidelines so that the trial provided an opportunity to estimate costs of real-life ART care in an urban setting. We analysed the resource use and costs of ART in both the routine health services and with the added intervention within REMSTART [17].

### Study settings

The REMSTART trial was implemented in Dar es Salaam, Tanzania, and Lusaka, Zambia, but the cost study was restricted to Tanzania for logistical reasons. Dar es Salaam has an estimated population of 4.4 million people [18] and an HIV prevalence among 15–49 year old of 6.9% [19]. The city is divided into three districts—Kinondoni, Ilala and Temeke. The trial was implemented at three government-run primary care health centres, one in each district (Tandale in Kinondoni, Buguruni in Ilala and Mbagala Rangi Tatu in Temeke).

In Tanzania, HIV is managed largely from primary care clinics. National guidelines at the time of the study were that patients presenting with a diagnosis of HIV-infection should have beeen offered ART if they had a CD4 count ≤ 350 cells/mm^3^ or if they were at WHO clinical stages 3 or 4 [20]. Follow-up clinic visits were initially every month and then two-monthly, when the patient was considered stable on therapy according to the clinician’s assessment. The REMSTART trial was restricted to patients who presented with advanced disease because they have very high rates of mortality [21–24]. Initially, patients with CD4 count <100 cells/mm^3^ were enrolled, but this criteria was changed subsequently to enrol patients presenting with < 200 cells/mm^3^, in order to speed up recruitment [17].

**Fig 1:**
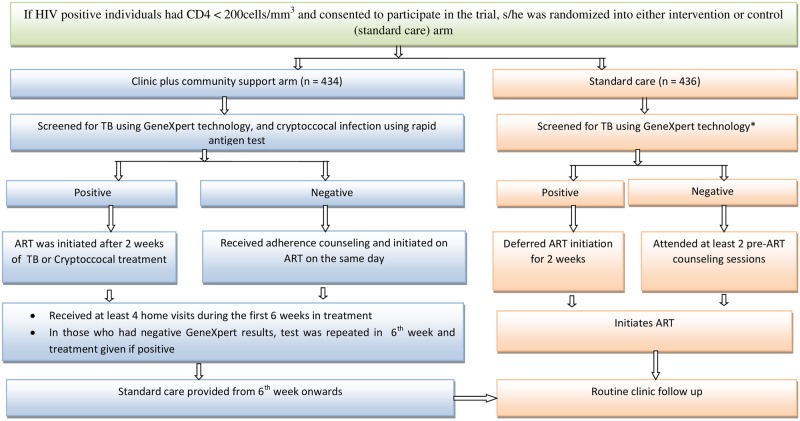
Pathway of care for HIV-infected individuals presenting in with CD4 count < 200 cells/mm^3^ in intervention and standard care arms in Tanzania. *Under standard care, TB screening is done using smear microscopy examination or chest x-ray. For this study, Xpert screening was used in both arms at baseline.

Participants were individually randomised [17] to receive either routine care or routine care plus additional clinic and community-based services (the REMSTART intervention). All participants (in both arms) were screened for tuberculosis (TB), irrespective of symptoms and tested for TB using GeneXpert^®^MTB/RIF assay (Cepheid, Sunnyvale, USA) (hereon referred to as Xpert) and ART was initiated rapidly where possible. Fig 1 shows both the pathway for and services provided to individuals in intervention and standard care arms. At the time of the trial implementation, Xpert was not part of standard care but all particpants were tested for TB using Xpert because it had been endorsed by the WHO as a screening tool for TB. Apart from screening for TB using Xpert test, study partcipants in the routine care arm were managed according to the standard of care. The clinic plus community support arm received the following additional services: i) screening for cryptococcal infection using serum cryptococcal antigen combined with pre-emptive fluconazle therapy for patients testing antigen positive; ii) weekly home visits for the first 4 weeks on ART by trained lay workers to provide adherence support; and (iii) re-screening for tuberculosis using the Xpert test at 6–8 weeks after ART initiation in participants in whom tuberculosis was not diagnosed at enrolment [17] (Fig 1).

### Data collection

Participants were recruited from February 2012 to September 2013, and each participant was followed for up to 1 year. Resource use and costs were measured from a provider’s perspective using a micro-costing approach. Health service resource use by the patient was tracked in trial records over a one year period for both patients under routine care and those in the clinic plus community plus support arms. This included number of clinic visits made by the patient, layworker home visits, diagnostic screening and treatment and inpatient costs. Cost data were collected as described in the supporting information (S1 Table) which lists the data collected and methods of collection at each of the sources. Costs were classified as recurrent or capital.

#### Allocation of costs

The time medical staff spent on different types of clinic visits was estimated using patient observations conducted over a one week period and personnel diaries self-completed by individual staff. Clinic visits were categorised as follows: i) a visit during which a clinical examination and CD4 count testing were done, ii) a visit during which the patient was assessed for ART eligibility, iii) a visit in which a patient self-referred to the clinic and was seen by clinician (sick visit), (iv) a visit during which a patient was clinically re-assessed following ART initiation and CD4 count testing was repeated, and v) a routine visit for the collection of drugs alone. Staff time spent on other activities was allocated to each visit in proportion to the daily patient contact time derived from personnel diaries. Such activities included morning report meetings, tea and lunch breaks, cleaning and preparing daily reports. As the clinic provides dedicated paediatric care on one day per week, 80% (4 out of 5 working days) of the shared costs were allocated to adult care. These costs were then divided by the average number of adult visits per month to obtain the cost per patient visit. Personnel time for laboratory work and lumbar punctures was derived from staff interviews. The laboratory equipment-related cost was calculated per test by dividing the annualised cost by the number of tests done. Research costs were excluded.

Costs incurred outside the clinic, other than laboratory costs, such as administrative costs, were not available for this study. Administrative costs reported elsewhere in resource limited settings ranged between 3% and 15.5% of the total costs of care [25,26]. Thus, we assumed an administrative cost of 10% of the clinic visit costs.

#### Cost valuation

All prices were converted to 2012 prices using the GDP deflator [27] and converted to US dollars (US$) (Exchange rate: 1US$ = 1,573.70 Tanzanian shillings -Tzshs) [28]. The annualization of capital costs, including training, was done using a discount rate of 7.8% equal to the 2-year Tanzanian government bond (at the time of calculation, June 2014) [29]. The annualization is the process of spreading the current purchase price of each item of equipment across its working life, taking into account its opportunity cost. The expected useful life for equipment varied from 3 to 15 years depending on the item [30]. Training of the layworkers was estimated to have a life span of 3 years.

### Analysis

Total and average resource use and costs per patient were estimated by month, quarter and year using time-series commands in Stata 13.0 (StataCorp). Costs were calculated separately for the routine care arm and then for the intervention arm. The denominator for the mean costs at all time points is all randomised patients rather than the those patients who were still alive at each time point.

#### Generalised linear regression analyses

We calculated a mean cost per patient (for 1 year of treatment) and used univariate and multivariate linear regression to explore the influence of study site [30], severity of illness (level of CD4 count and WHO clinical stage), marital status, level of education, gender and age [31–34] on the mean cost of HIV/AIDS care among those who received standard care.

#### Sensitivity analyses

To account for uncertainty in some of the key variables, univariate sensitivity analyses were done in which parameters were varied one at a time using values given in the supporting information (S2 Table). The discount rate was varied to ensure comparability with other studies [35]. Prices for ART, reagents and the Xpert cartridges were varied to explore the impact of any changes in the international market prices. Costs incurred above the clinic level were varied to reflect the uncertainty in the baseline estimate. In addition, the analysis was run replacing the cost of the Xpert with a cost for smear microscopy, given the limited use of the Xpert to date in sub-Saharan Africa. The transportation costs of Xpert sputum samples (from the clinics to the central tuberculosis reference laboratory where Xpert machines are located) are critical to cost-effectiveness of a programme [25], therefore this transport cost was varied to reflect any uncertainty in replicating the trial transport mechanism. Further, the number of home visits per day by a lay worker was varied from the base case scenario to a more likely work load scenario in a real life setting in which more home visits would be conducted. Detailed methods for the sensititivity analysis can be found in the supporting information (S2 Table). The uncertainty analyses were based on most likely, minimum and maximum values.

#### Ethics statement

The study was nested within a large trial (ISCRTN 20410413) which was approved by the ethics committee of the London School of Hygiene & Tropical Medicine, the Ethics and Research Science committee in Zambia, and the National Health Research Ethics Sub-Committee in Tanzania. All study participants were older than 18 years and each provided a written informed consent.

## Results

### Characteristics of the study participants in Tanzania

A total of 870 individuals, 436 in standard care arm and 434 in the intervention arm, were enrolled in the study. The median (IQR) age of patients was 38 [31,44] years, 62% were female, and the median (IQR) CD4 cell count at enrolment was 52 [20,89] cells/mm^3^ (Table 1). Overall, the median (IQR) length of follow up was 333 (347, 365) days. The majority of participants were initiated on TDF-containing (60%) or AZT-containing (36%) ARV regimens. Overall, after a year of follow up, 684 (78.6%) participants were alive and receiving care at the study clinics, 153 (17.6%) had died, 8 (0.9%) had withdrawn from the study and survival status for 25 (2.9%) participants was unknown. Sixty six of 153 (43.1%) participants who had died were from the intervention arm. Overall, 22 (5%) of the 434 participants screened for cryptococcal antigen in the intervention group tested positive for serum cryptococcal antigen.

**Table 1:** Characteristics, HIV disease progression and ART treatment regimen of for study participants (HIV patients presenting in the advanced stages of HIV-infection—CD4 <200cells/mm^3^), in Dar es Salaam, Tanzania.

Characteristics	Study sites			
	Buguruni	Tandale	Mbagala	All sites
Sample size	196	280	394	870
Standard (control) arm	99	140	197	436
Intervention arm	97	140	197	434
**Sex**				
Male n(%)	58 (29.6)	105 (37.5)	164 (41.6)	327 (37.6)
**Age at enrolment** mean (SD)	39.1 (10.4)	37.4 (8.6)	39.2(9.9)	38.6 (9.7)
CD4 cells/mm^3^, median (min, max)	52 (2, 189)	57.5 (1, 199)	47 (1, 199)	52. (1, 199)
**ART**^a^ **regimen initiated at enrolment n(%)**				
d4T-containing regimen	0 (0.00)	0 (0.00)	10(2.54)	10 (1.15)
AZT-containing regimen	68 (34.69)	146 (52.14)	100 (25.38)	314 (36.09)
TDF -containing regimen	125 (63.78)	127 (45.36)	269 (68.27)	521 (59.89)
ABC -containing regimen*	2 (1.02)	5 (1.79)	1 (0.25)	8 (0.92)
Did not initiate on ART	1 (0.51)	2 (0.71)	14 (3.55)	17 (1.95)

^a^ART—Antiretroviral Therapy; ABC—abacavir; TDF—tenofovir disoproxil fumarate AZT—zidovudine; d4T –stavudine

*ABC is the nucleoside reverse transcriptase inhibitors (NRTIs) used as the second line drugs for adult and adolescents in Tanzania.

### Unit costs for different components of HIV care

Table 2 presents estimates of unit costs, at each clinic, for the different components of ART services. The mean clinic cost for outpatient visits ranged between US$ 5.76 for a drug collection visit without clinical assessment to US$ 6.74 for a visit that included a clinical assessment and a CD4 count test. The cost of a home visit conducted by a lay-worker was $14.74. The cost of diagnostic tests ranged between US$19.07 for an Xpert test and US$ 0.31 for a creatinine test. The novel CrAg test was estimated to cost $3.96 per participant.

**Table 2:** Unit costs (in 2012 US$) of the different components of ART services for patients initiating treatment with CD4 count <200 cells/mm^3^ in Dar es Salaam, Tanzania.

Variable	Unit cost (2012 US$) (per patient)			
	Buguruni	Mbagala	Tandale	Mean cost for all sites
**Costs for clinic outpatient visits under ART programme**				
Initial visit (assessed by clinician, blood for CD4 test taken)	6.61	6.13	7.48	6.74
ART eligibility assessment visit	6.04	5.10	6.96	6.04
Sick visit e.g. patient self-referred and seen by clinician*	6.04	5.10	6.96	6.04
6-monthly clinic (reassessed by clinician, blood sample for CD4 test taken)**	6.61	6.13	7.48	6.74
Routine follow up visit e.g. drug pick up and not seen by clinician	5.89	4.75	6.65	5.76
**Costs of diagnostic tests in addition to outpatient visit costs**				
CD4 count test	15.83	14.75	16.72	15.76
Alanine aminotransferase (ALAT) test	0.88	0.84	0.91	0.88
Creatinine test	0.32	0.28	0.35	0.31
Haemoglobin (Hb) test	0.83	0.86	0.91	0.87
Random blood glucose (RBG) test	0.85	0.81	0.89	0.85
Venereal disease reference laboratory (VDRL) test	1.88	1.88	1.89	1.89
Pregnancy test	0.53	0.53	0.53	0.53
Full Blood Count (FBC) test	2.06	1.79	2.29	2.04
Serum cryptococcal meningitis (CRAG) test	5.00	2.68	4.19	3.96
**Costs of the service components performed outside the clinic**				
Xpert test^b^				19.07
Collection and processing of cerebral spinal fluid^c^				16.29
Chest x-ray^d^				3.18
Lay worker visit to the patients home				14.74
**Information obtained from the literature**				
Smear microscopy test				1.81
Sputum culture (Lowenstein Jensen) for TB [25]				6.84
ART regimens[15] (costs for daily dose)				
d4T + 3TC +NVP				0.16
d4T + 3TC + EFV				0.28
AZT + 3TC +NVP				0.31
AZT + 3TC + EFV				0.61
TDF + 3TC + NVP				0.32
TDF + 3TC + EFV				0.60
ABC +3TC + NVP				0.13
ABC +3TC + EFV				0.76
TDF + FTC + EFV				0.60
TDF + FTC + NVP				0.48
TDF + FTC + ABC				0.84
Fluoconazole per a dose of 10 weeks				4.80
Cotrimoxazole per daily dose				0.02
District Hospital inpatient bed day[36]				26.45
Regional Hospital inpatient bed day[36]				24.72
Tertiary Hospital inpatient bed day^e^				24.01
TB treatment (6 monthly dose)				225

* Involves similar activities and therefore unit costs were assumed to be the same as for the ART eligibility assessment visit

** Involves similar activities and therefore unit costs were assumed to be the same as for the initial visit.

^b^Performed at the Central TB laboratory

^c^Done at Muhimbili National Hospital

^d^performed at the respective district hospital

^e^Costs for tertiary hospital were not available; we therefore assumed these costs were the same as those of the regional hospital

Costs for personnel and non-medical material plus supplies constituted more than 60% and 13% respectively of the unit costs for out-patient clinic visits, excluding diagnostic procedures. Costs of the reagents for CD4, CrAg and Xpert were 85%, 99% and 70% of the unit costs for these tests (see S3 Table). There was little variation between the clinics in the breakdown of costs (Table 2).

### Resource utilization

Table 3 summarizes the information on resources used by the study participants. Seventeen individuals (7 randomised to standard care and 10 to the intervention arm) of 870 (2%) did not start ART. Twelve of these individuals died soon after presentation, 1 was lost to follow up and 4 withdrew from the study before starting ART.

**Table 3:** Unit costs (in 2012 US$), average quantity of resources utilized over 12 months of treatment and mean annual cost per patient, for patients initiating treatment with CD4 count <200 cells/mm^3^ in Dar es Salaam, Tanzania.

Item	Unit cost (US$2012)	Mean? resource utilisation per person		Cost per patient (US$2012)		Difference (Intervention—control)
		Intervention	Standard care	Intervention arm	Standard care	
**Out patients visits**						
Initial visits	6.74	1.05	1.03	7.08	6.94	0.13
ART eligibility assessment visits	6.04	1.00	1.00	6.04	6.04	0.00
Sick visits	6.04	0.17	0.10	1.03	0.60	0.42
6-montly clinic review	6.74	0.89	0.86	6.00	5.80	0.20
Routine follow up visits	5.76	4.43	4.41	25.52	25.40	0.12
CD4 count test	15.72	1.60	1.56	25.15	24.52	0.63
ALT test	0.88	1.20	1.1	1.05	0.97	0.09
Creatinine test	0.31	0.77	0.74	0.24	0.23	0.01
Hb test	0.87	1.33	1.17	1.16	1.02	0.14
VDRL test	1.89	0.07	0.06	0.13	0.11	0.02
Pregnancy test	0.53	0.07	0.08	0.04	0.04	-0.01
Second Xpert test	19.07	0.88	0.00	16.78	0.00	16.78
Chest x-ray	3.18	0.06	0.05	0.17	0.16	0.02
CrAg test	8.43	1.00	0.00	8.43	0.00	8.43
Lay worker visit to the patients home	14.74	2.89	0.00	42.60	0.00	42.60
Collection and processing of cerebral spinal fluid	16.27	0.02	0.00	0.37	0.00	0.37
Days on ART drugs	0.42	323.12	213.60	135.71	131.71	4.00
Days on Co-trimoxazole	0.02	270.22	265.19	5.40	5.30	0.10
Days of hospital admissions	26.43	3.79	4.08	100.13	107.78	-7.66
Number of people put on fluconazole	4.8	0.09	0.00	0.43	0.00	0.43
**Mean cost per patient during the first three months and one year in treatment (in 2012US$)**						
First three months in treatment, mean (95% CI)				166 (161–171)	107 (101–112)	59 (52–66)
One year in treatment, mean (95% CI)				331 (319–344)	265 (254–275)	67(50–83)

Of 436 patients in the standard care arm, 270 (62%) completed one year of follow up, which was defined as those individuals who were alive and receiving care at one of the study clinics, 66 (15%) had died, 60 (13.8%) did not return to a study site at 12 months, 37 (8.5%) withdrew from the study and information for 3 participants was missing. Overall, the study participants in the standard care arm spent 308.9 person years in ART care, equivalent to a mean follow up on ART of 0.71 year per patient. Of the total time on treatment, 62% and 37% was spent on ART regimens containing TDF and AZT, respectively; and 1% was spent on other regimens. Thirty of 429 (7%) patients who initiated on ART substituted at least one drug in their regimen during the one-year period of follow up. Patients in the standard care arm were on co-trimoxazole for a mean of 0.73 years during the study period.

In the intervention arm, 289/434 (66.6%) patients completed one year of follow up. Of those who continued to receive care from the study clinics, 51/434 (12%) had died, 36/434 (8%) did not return to a study clinic, 56/434 (13%) withdrew from the study and information for 2 participants was missing. Overall, the study participants in the intervention arm spent 316.4 years in ART care, equivalent to a mean follow up on ART of 0.73 year per patient. Of the total time on treatment, 64% and 34% was spent on ART regimens containing TDF and AZT, respectively, and 2% was spent on other regimens. Forty-four of 388 (11%) patients who were initiated on ART substituted at least one drug in their regimen during the one year period of follow up. Patients in the intervention arm were on co-trimoxazole for a mean of 0.74 years during the study period. Compared to patients in standard care, patients in the intervention arm had significantly more sick clinic visits (p = 0.001), alanine aminotransferase tests (p = 0.039), and Hb tests (p = 0.0013).

### Average costs per patient for routine care

**Fig 2:**
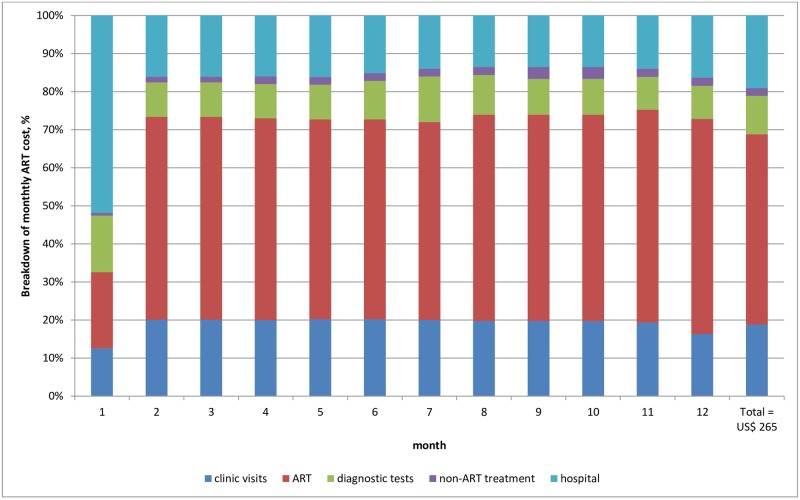
The cost breakdown of ART services by month for patients initiating treatment with CD4 count <200 cells/mm^3^ in Tanzania for standard care.

**Fig 3:**
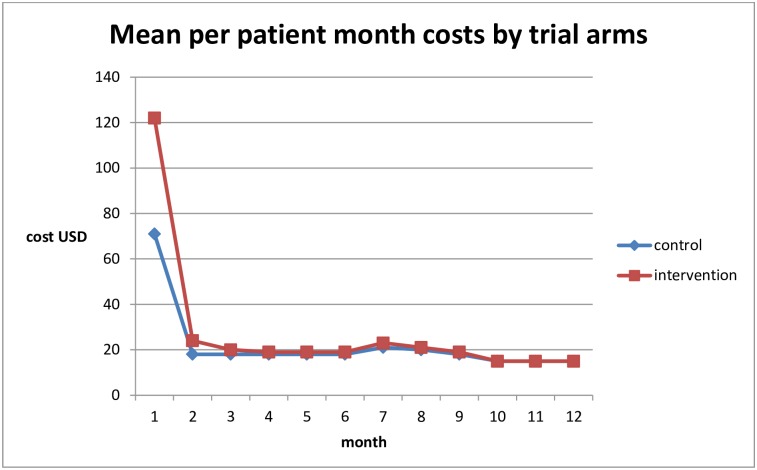
Mean cost (in USD 2012) per patient month for patients initiating ART treatment with CD4 count <200 cells/mm^3^ in Tanzania for both standard care (control) and the REMSTART trial intervention arms.

The mean cost per patient year was US$ 265 (95% CI 254–275) in the standard care arm (Table 3). ART drugs, clinic visits, hospital admission, diagnostic tests and non-ART drugs constituted 50%, 19%, 19%, 10% and 2% of the total cost per patient year respectively (Fig 2). Initial high costs of US$71 per patient month in the first month fell to US$ 30 per patient month at 12 months (see Fig 3). The cost per patient was US$107 (101–112) in the first quarter, after which it fell and stabilised at between $45 and $59 per quarter.

The mean annual cost of ART per participant varied from $34.89 for stavudine-based regimens to $140.60 for tenofovir-based regimens. Costs of co-trimoxazole prophylaxis were just over $5 per patient per year. Routine follow up visits were more common than other visits and consequently accounted for a higher cost than other visits. CD4 count and the Xpert testing were the most expensive laboratory tests, with serum CrAg testing costing just under $4 per test per patient. Hospital admission costs exceed $43 per admission and comprised a mean of 4.87 days of inpatient care per patient.

Table 3 also compares the costs in the standard and intervention arms. The cost of adding CrAg screening, home visits by lay-workers and a second Xpert test, as implemented in the clinic plus community support arm of the trial, were US$ 8.43, US$42.60 and US$16.78 per patient, respectively (see Table 3). The incremental costs per patient of the full intervention to the health service over the first three months was US$ 59 (p<0.001; 95%CI 51.5–66.5) and over a one year period was US$ 67(p<0.001; 95%CI 50.0–83.2). This is equivalent to a 55% (95%CI 50.7%–59.2%) increase in the mean cost of care over the first three months and a 25% (95%CI 19.7%–30.2%) increase over one year of follow up.

### Sensitivity analyses

The cost of care was robust to changes in the variables explored in the sensitivity analysis, except to changes in the average number of home visits made by lay worker in a day, costs of clinic visits involving a CD4 test and the cost of antiretroviral drugs. A 25% decrease in the price of antiretroviral drugs led to a reduction in the mean costs per patient year of 10.6%, and an increase in the number of home visits from 1.4 to 4 per day resulted in a 22% reduction in the additional cost for the intervention component during the first three months, and a 12% over a one year period of follow up, whereas, an increase of costs for clinic visits involving a CD4 test, by 50%, during the first three month of ART led to an increase in the mean costs per patient year of 22.7%. A decrease in this by 50% resulted in a reduction of per patient cost by 11.4%, (Table 4).

**Table 4:** Results of the sensitivity analyses exploring the impact of uncertainty in key variables on the average cost estimates for patients initiating ART treatment with CD4 count <200 cells/mm^3^ in Dar es Salaam, Tanzania.

Arm	Parameters varied	Varied values		US$ (% divergence from base case)			
				1^st^ three month		Per Patient Year	
		Minimum	Maximum	Minimum	Maximum	Minimum	Maximum
**Standard care**	Base case*			106.9	106.9	264.7	264.7
	Discount rate	1%	12%	106.3 (0.6)	107.3 (0.4)	263.4 (0.5)	265.8 (0.4)
	Administrative costs	3%	15.5%	104.8 (2.0)	108.4 (1.4)	262.6 (0.8)	266.3 (0.6)
	Costs for supporting staff	-50%	+50%	104.1 (2.6)	112.5 (5.2)	261.5 (1.2)	271.2 (2.4)
	Costs for clinic visits involving a CD4 test	-50%	+50%	94.7 (11.4)	131.2 (22.7)	259.9 (1.8)	274.2 (3.6)
	Costs for clinic visits involving seeing a clinician	-50%	+50%	103.7 (3.0)	113.2 (5.9)	262.3 (0.9)	269.5 (1.8)
	Costs for routine follow up	-50%	+50%	106.8 (0.1)	124.1 (16.1)	255.7 (3.4)	268.9 (1.6)
	Costs for Xpert	-5%	-25%	106.0 (0.8)	102.4 (4.2)	263.9 (0.3)	268.1 (1.3)
	Price for Xpert cartridge	-5%	-25%	106.4 (0.5)	104.2 (2.5)	264.2 (0.2)	262.6 (0.8)
	Costs for transport for Xpert sputum sample	0%	-50%	106.4 (0.5)	106.6 (0.3)	264,2 (0.2)	264.4 (0.1)
	Price of ARV	-5%	-25%	104.8 (2.0)	96.3 (9.9)	259.1 (2.1)	236.6 (10.6)
	Costs for sputum smear microscopy	0%	100%	106.9 (0.0)	105.5** (1.5)	264.7 (0.0)	262.3** (0.9)
**Intervention**	Base case*			165.9	165.9	331.4	331.4
	Price for CRAG reagents	-5%	-25%	165.9 (0.0)	165.9 (0.0)	331.4 (0.0)	331.4 (0.0)
	Monthly salary for lay workers	US$177	US$383	156.6 (5.6)	165.9 (0.0)	325.1 (1.9)	334.4 (0.9)
	Monthly income (salary & benefits) for lab technician for processing the cerebral spinal fluid	US$379	US$397	165.7 (0.1)	166.1 (0.1)	331.4 (0.0)	331.5 (0.0)
	Number of home visits by lay-worker	1	4	174.1 (4.9)	129.2 (22.1)	335.4 (1.2)	290.5 (12.3)

*Does not include costs for smear tests

**Does not include costs for Xpert test

### Regression

Table 5 reports on the results of the univariate and multivariate regression analyses of factors that could influence health service costs in the standard care arm. None of the factors was significantly associated with health service costs.

**Table 5:** Univariate and multivariate regression (Generalized Linear model) analyses of factors that could influence ART service costs (in 2012 US$) for patients initiating treatment with CD4 count <200 cells/mm^3^ in Dar es Salaam, Tanzania.

Independent variable	Results of the Generalized Linear regression analyses (N = 870)			
	Univariate analysis	F-test	Multiple regression¥	F-test
	*Coefficient (95% CI)	p-value	Coefficient (95% CI)*	p-value
**Age (reference is “Age less than 45years”)**		0.5415		0.2702
**≥ 45 years**	**7.71 (-17.03–32.44)**		14.67 (-11.38–40.66)	
**Level of CD4 count at enrolment (reference is “CD4 <25cells/mm**^**3”**^		0.3698		0.3984
CD4 25–49cells/mm^3^	20.57 (-10.75–51.90)		20.83 (-10.94–52.59)	
CD4 ≥50cells/mm^3^	13.64 (-10.37–37.65)		12.17 (-12.20–36.54)	
**WHO clinical stage at enrolment (reference is “WHO stage 1 or 2”)**				0.9154
WHO stage 3 or 4	-4.94 (-32.50–22.62)	0.7254	-1.54 (-29.98–26.90)	
**Sex**				0.1168
Female	11.70 (-10.08–33.49)	0.2924	18.70 (-4.67–42.06)	
**Level of education (reference is “no formal education”)**		0.4358		0.3954
Primary	19.45 (-10.26–49.16)		20.98 (-9.78–51.73)	
Secondary and above	17.79 (-20.78–56.37)		21.36 (-18.35–61.07)	
**Clinic (reference is (“Buguruni clinic”)**		0.1036		0.1284
Tandale clinic	18.46 (-10.53–47.45)		22.63 (-7.15–52.42)	
Mbagala clinic	-7.89 (-35.08–19.31)		-1.88 (-29.92–26.17)	
Marital status (reference is “married or living with someone”		0.9545		0.8574
Widowed/separated/divorced	-2.45 (-26.08–21.18)		-5.60 (-29.82–19.70)	
Never married	-4.33 (-34.44–25.78)		-7.91 (-39.33–23.52)	

^¥^All variables included simultaneously in a single model

*Mean difference in health service costs (in US$) compared to the reference group

## Discussion

The costs of antiretroviral therapy in Africa have fallen remarkably. Nonetheless, as this study demonstrates, the average cost of care remains high at nearly $300 per patient per year and over half of this expenditure was on drugs. Using a micro costing approach, this study has calculated the average cost per patient year of ART as well as the incremental cost of an innovative intervention able to reduce mortality in ART patients. The analysis shows how costs fall over time and that ART drugs continue to take up the largest share of ART costs. We did not calculate the costs incurred by patients to access care, but previous studies have found these to be remarkably high [37]. It is evident, that HIV continues to be a substantial burden in Africa.

The costs of the clinic plus community support intervention, which mostly targeted the first month of ART, and which reduced all-cause mortality substantially [17], were about 25% higher than the standard treatment arm, when calculated over a 12-month period. The analysis by month and by quarter confirm that the increase in cost is only associated with the initial stage of treatment and as such do not have a major impact on the lifetime costs of ART, providing a strong argument for investment given the likely reduction in mortality that was demonstrated by the trial. Although this study was done as part of a trial, it was integrated into the health system, with patients managed by routine health care staff. Thus the costs presented are likely to be typical of urban clinics in Tanzania among HIV-infected individuals presenting with advanced disease. However, a formal economic evaluation is needed to determine if and to what extent this intervention is cost-effective compared to standard care.

The cost of a single home visit was nearly $15, just over twice the cost incurred by health services when a patient visits the clinic for routine care and similar to the cost of a CD4 count test. The cost of home visits may be lower in real life settings if lay-workers can visit a greater number patients per day. In our trial, most patients (in both arms) were initiated on ART on their second visit to clinic, within a median of about two weeks [17], because we introduced expedited initiation of antiretroviral therapy, whereas in most African HIV programmes, patients are required to attend 3–4 times before ART is initiated. Our study demonstrates that these early pre initiation visits are probably unnecessary and that it would be better to provide additional support after the patient has started antiretroviral therapy.

Another striking finding was that the care delivered by the clinics differed substantially from guidelines. The mean number of clinic visits involving a clinician was less than 2 visits per patient during the first year on antiretroviral therapy, compared to the recommended 6 visits [20,32,38]; and CD4 count and safety blood testing were done much less frequently than recommended. It is possible that this was because of shortages of staff and reagents, that patients were not returning for required visits or that clinics felt that these visits were not necessary.

The average cost per patient year of standard care in this study was marginally higher than that reported from Zambia [11] and Nigeria [12], although the Nigeria study excluded overhead costs. In accordance with our findings, in other studies conducted across Africa, antiretroviral drugs have been the most expensive component of care [9–14,16]. We found that reducing the price of antiretroviral drugs by 25% lowers the annual cost per patient by 11%; this highlights the importance of international efforts to negotiate reductions in antiretroviral drugs prices.

Initially, our study enrolled patients with CD4 count <100 cells/mm^3^, but this criteria was changed subsequently to enrol patients presenting with <200 cells/mm^3^, in order to speed up recruitment. This change affected both trial arms equally. and presumably was not felt to compromise the interpretation of the study from a clinical standpoint. The univariate and multivariate regression analyses of factors that could influence health service costs, the level of CD4 count was not significantly associated with health service costs, so it is unlikely this change had any effect on the cost estimate.

Our study was conducted among urban patients with advanced disease. Whether the findings are generalizable to rural patients, who are usually more geographically scattered and less mobile, or among patients initiating ART in the early stages of HIV-infection needs further research. We used university graduates as the lay workers and it is not clear if using lay-workers with fewer qualifications would have the same effect. This needs further research as it may be difficult to scale-up the programme using university graduates.

## Conclusion

Micro costing approaches provide a way to obtain an in-depth understanding of cost structures and cost variation between individuals as well as over time. This study confirms that despite price reductions, antiretroviral drugs constitute half of the cost of care for people newly starting on ART and confirms that monthly costs of ART decline after the first month of treatment. The added costs of the clinic plus community support intervention, which mostly targets the first month, and which substantially reduced all-cause mortality, represented only a small increase in life time ART costs, suggesting this important innovation in ART could be an affordable approach to improving HIV care in resource constrained settings.

## Supporting information

S1 Table Information on resources use and prices by cost centre. (DOCX)

S2 Table Reasons for uncertainty. (DOCX)

S3 Table Recourse utilization by the components of ART. (DOCX)
