# Supplementary material for: The costs of providing antiretroviral therapy services to HIV-infected individuals presenting with advanced HIV disease at public health centres in Dar es Salaam, Tanzania: Findings from a randomised trial evaluating different health care strategies
Source: PLoS One. 2017 Feb 24;12(2):e0171917. doi: 10.1371/journal.pone.0171917 (PMC5325220; doi:10.1371/journal.pone.0171917)
Supplement: S1 Table — (DOCX) [file pone.0171917.s001.docx]

S1 Table. Information on resources use and prices by cost centre

| Name of the cost center | **Costing data collected** | **Source of resources and quantity used** | **Source of price information** |
| --- | --- | --- | --- |
| Clinic | 1. Recurrent costs |  |  |
|  | Personnel | Three approaches were used to collect information on staff time:   - Observing the time patient spends with each cadre as s/he moves from one service delivering point to another. This was done for one week per clinic. Stop watch was started when patients enters in the provider’s room and stopped when patient leaves the room - Interviewing clinic laboratory personnel on the time spent in collecting and processing samples - Each staff was asked to fill in a form showing the way s/he spends his/her time from morning (when clinic opens) to evening (when clinic closes) from Monday to Friday. | Salary and benefit information from:   - clinic administration offices - Interviewing an individual staff |
|  | Medical and non-medical materials and supplies | Type and average monthly quantity for each item obtained from the clinic administration office. | MSD whenever possible or/and from other suppliers. |
|  | Drugs for HIV/AIDS treatment | Interview with the clinic pharmacist and review of the clinic pharmacy documents. | Drugs for OIs - MSD*  ART - Medicins Sans Frontieres (MSF)^a^ |
|  | Medical supplies specific to patients | Information on different inputs and their quantities required for patients: diagnostics (e.g CD4 count); monitoring (e.g ALAT**, creatinine and Haemoglobin) and screening for cryptoccocal infection using CRAG was obtained from the laboratory in-charge. | - Prices for CD4, ALAT**, creatinine and Hb reagents - MSD. - CRAG test - consultations with experts in the area of management of cryptoccocal meningitis. Its cost at delivery was obtained from the local distributor in Tanzania. |
|  | Utility (electricity) | Average monthly diesel consumption for generator obtained from the MDH procurement offices; and that of mains electricity utilisation was obtained from electricity bills from the respective municipal offices. | - Average price of a litre of diesel from local filling stations for fuel electricity bills for unit price of electricity |
|  | Transport operating costs and other transport costs | Monthly average fuel consumption and insurance premium was obtained from the MDH procurement office for motorbike and vehicle. | Average price of a litre of diesel from local filling stations. |
|  | Medical supplies specific to patients | Equipment required for patient diagnostics (e.g., CD4 count) and monitoring (e.g., ALAT, creatinine and Haemoglobin) obtained from the clinic laboratory in-charge | Replacement unit price for machines for CD4, ALAT, creatinine and Haemoglobin - MDH procurement offices |
|  | 1. Capital costs |  |  |
|  | Building | Area covered by the clinic, health centre and TB clinic was obtained from the facility administration offices or measured using a tape measure | Market rental price per m^2^ - local estate agencies |
|  |  |  |  |
|  | Equipment, furniture and Other capital (office equipment) | A list of equipment, furniture and other capital costs available in each clinic was obtained from the respective clinic administration office. | Replacement unit prices were obtained from MSD or MDH procurement offices whenever possible, and from other suppliers recommended by MDH if not available. |
|  | Transport | Transport details were obtained from the clinic administration offices. | Replacement unit prices of motorbike and vehicle were taken from MDH procurement offices |
|  |  |  |  |
| Muhimbili National Hospital | 1. Recurrent costs |  |  |
|  | Personnel | Two approaches were used to collect information on personnel involved in performing lumbar puncture (LP) to collect cerebral spinal fluid (CSF) and processing the CSF :   - Observation when the LP was being performed, using a stopwatch. - Interviewing the laboratory technician for the time spent to prepare and process one sample of the CSF | Staff salary and benefits - interviewing the respective staff |
|  | General medical materials and supplies for LP | Carrying out LP: type and quantity of materials obtained by interviewing the nurse who prepares the LP tray  Processing of CSF: type and monthly average quantity of resources specific to CSF used per month obtained from the offices of the hospital laboratory. Quantity per test by dividing by number of the CSF processed per month | Unit price was obtained from MSD or suppliers recommended by the in-charge of the MNH laboratory. |
|  | 1. Capital costs |  |  |
|  | Incubator and microscopy | Type and capacity of each piece of equipment obtained from the MNH laboratory in-charge | Suppliers. |
|  |  |  |  |
| Central and Reference Tuberculosis Laboratory | 1. Recurrent costs |  |  |
|  | Personnel | Staff time spent for preparing and processing of the GeneXpert test was obtained by interview with the laboratory technician involved | Salary and benefit information by interviewing the respective staff |
|  | Medical supplies specific to GeneXpert test | Information on inputs and quantities required for each test obtained from the laboratory technician. | Supplies - the manufacturer^b^ |
|  | Medical and non-medical materials for GeneXpert | Inputs and quantities required for each test identified through interviewing laboratory technician | The MSD whenever possible or from other suppliers. |
|  | 1. Capital costs |  |  |
|  | Equipment | List of equipment required obtained from technician | GeneXpert machine - the manufacturer^b^  Timer and thermometer - suppliers recommended by the CTRL in-charge. |
|  |  |  |  |
| National Institute for Medical Research, Muhimbili Medical Research Centre | Personnel  - layworkers  - runners | Terms of employment obtained from REMSTART trial records  Travel allowance obtained from REMSTART trial records  Communications allowance obtained from REMSTART records | REMSTART administrator’s office  REMSTART administrator’s office  REMSTART administrator’s office |
|  | Travel  Communication |  |  |
|  |  |  |  |
|  | 1. Capital costs |  |  |
|  | 1. layworker training | Number, length and frequency of training obtained from REMSTART trial records | REMSTART administrator’s office |
|  | Communication facilities | Type and number of mobile phone handsets obtained from RESMTART trial records | REMSTART administrator’s office |
|  |  |  |  |

^*^Medical Store Department ; **alanine aminotransferase ; ^a^MSF Access UTW, 16^th^ Edition 2013

^b^<http://www.cepheidcares.com/tb/index.php> (Accessed in March 2012)
