# Supplementary material for: The costs of providing antiretroviral therapy services to HIV-infected individuals presenting with advanced HIV disease at public health centres in Dar es Salaam, Tanzania: Findings from a randomised trial evaluating different health care strategies
Source: PLoS One. 2017 Feb 24;12(2):e0171917. doi: 10.1371/journal.pone.0171917 (PMC5325220; doi:10.1371/journal.pone.0171917)
Supplement: S2 Table — (DOCX) [file pone.0171917.s002.docx]

S2 Table. Reasons for uncertainty

| Parameter | Reasons |
| --- | --- |
| Discount rate | Rates used in most publications range between 3% and 6% [37] and the discount rate reported by the bank of Tanzania (in 2012) is 12% [38]. In this analysis we used the rate of interest for government bond [29]. |
| Administrative costs | We could not obtain information on administrative costs from the relevant offices so we assumed it was 10% of total costs of the clinic visits. However, the administrative costs reported elsewhere in resource limited settings range between 3% and 15.5% of the total ART costs. |
| Costs for supporting staff | The sites included in this analysis had an average of 4 data clerks per site. We assumed two data clerks would be sufficient. In making assumption, we also considered patients load in the clinic apart from the one enrolled in this study. |
| Costs for clinic visits | On average, patients had more than one clinic visit before treatment initiation and several others thereafter. We assumed the number of visits could be reduced to one for assessment and treatment initiation and fewer routine visits thereafter for stable patients. |
| Costs for transport for Xpert sputum sample | Sputum samples for TB diagnosis were shipped to the central laboratory for processing using Xpert technology. The Xpert technology is now being decentralized to districts hospital level, which are close to the HIV clinics, as results costs for samples shipment is expected to drop to less than half its current value. |
| Price for Xpert cartridge | The price for Xpert cartridge is expected to come down, as there have been international efforts to negotiate the prices down in areas where resources are scarce.  (www.finddiagnostics.org/about/what_we_do/successes/find-negotiated-prices/xpert_mtb_rif.html) |
| Price of ARV | We used ARV prices information from MSF. According to the MSF 2013 report, the prices of ARVs dropped sharply in 2010 [15]. Our assumption is that, these prices will decline further. |
| Costs for sputum smear microscopy | Under standard care, TB diagnosis is done using smear microscopy. For the REMSRAT trial [17], we used both smear microscopy and Xpert for TB diagnosis. Under normal circumstance, only one of these methods will be used. |
| Costs for lay workers | In the REMSTART trial [5], we used university graduates as the lay workers. We assumed we could use staff with lower qualifications as have been used in other programmes. |
| Costs for processing CSF | In the REMSTART trial [5], we used laboratory scientists to process the CSF for the trial patients. Under standard care, a laboratory technician does this. Monthly salary and benefits for the lab technicians are lower than those of laboratory scientists. |
| Home visits | The average number of home visits per day per lay-worker used at the base case was 1.4. Since the trial was implemented in a city, we assumed a lay-worker could visit an average of 4 homes per day. |
