# Supplementary material for: The costs of providing antiretroviral therapy services to HIV-infected individuals presenting with advanced HIV disease at public health centres in Dar es Salaam, Tanzania: Findings from a randomised trial evaluating different health care strategies
Source: PLoS One. 2017 Feb 24;12(2):e0171917. doi: 10.1371/journal.pone.0171917 (PMC5325220; doi:10.1371/journal.pone.0171917)
Supplement: S3 Table — (DOCX) [file pone.0171917.s003.docx]

| S3 Table. Recourse utilization by the components of ART | | | | | | | | | | | | | |
| --- | --- | --- | --- | --- | --- | --- | --- | --- | --- | --- | --- | --- | --- |
|  | % TOTAL COST | | | | | | | | | | | | **TOTAL COSTS** |
| Components of ART services | PERSONNEL | NON-MEDICAL MATERIALS & SUPPLIES | MEDICAL MATERIALS & SUPPLIES | TRANSPORT OPERATING | UTILITIES | BUILDING | TRANSPORT | EQUIPMENT | FURNITURE | OTHER CAPITAL | COMMUNI-CATION | TRAINING |  |
| Initial visits | 67.15 | 13.01 | 5.20 | 1.79 | 0.81 | 6.50 | 1.30 | 0.33 | 1.63 | 2.28 |  |  | 6.74 |
| ART eligibility assessment visits | 62.57 | 14.68 | 5.87 | 2.02 | 1.28 | 7.34 | 1.47 | 0.37 | 1.83 | 2.57 |  |  | 6.04 |
| Sick visits | 62.57 | 14.68 | 5.87 | 2.02 | 1.28 | 7.34 | 1.47 | 0.37 | 1.83 | 2.57 |  |  | 6.04 |
| 6-monthly clinic review | 67.15 | 13.01 | 5.20 | 1.79 | 0.81 | 6.50 | 1.30 | 0.33 | 1.63 | 2.28 |  |  | 6.74 |
| Routine follow up visits | 60.93 | 15.47 | 6.19 | 2.13 | 0.97 | 7.74 | 1.55 | 0.39 | 1.93 | 2.71 |  |  | 5.76 |
| CD4 count test | 0.32 | 0.00 | 85.41 |  |  |  |  | 14.28 |  |  |  |  | 15.76 |
| ALT test | 1.14 | 0.00 | 93.18 |  |  |  |  | 5.68 |  |  |  |  | 0.88 |
| Creatinine test | 3.13 | 0.00 | 81.25 |  |  |  |  | 15.63 |  |  |  |  | 0.31 |
| Hb test | 1.15 | 0.00 | 94.25 |  |  |  |  | 4.60 |  |  |  |  | 0.85 |
| VDRL test | 0.53 | 0.00 | 99.47 |  |  |  |  | 0.00 |  |  |  |  | 1.89 |
| Pregnancy test | 1.89 | 0.00 | 98.11 |  |  |  |  | 0.00 |  |  |  |  | 0.53 |
| Xpert test | 19.76 | 5.50 | 70.67 |  |  |  |  | 4.06 |  |  |  |  | 19.01 |
| Serum cryptoccoccal antigen (CrAg) test | 0.22 | 0.00 | 99.78 |  |  |  |  | 0.00 |  |  |  |  | 3.96 |
| Lumbar puncture | 70.10 | 0.00 | 29.59 |  |  |  |  | 0.31 |  |  |  |  | 16.29 |
| Lay worker visit to the patients home | 91.98 |  |  | 0.34 | 14.74 |  | 7.67 |  |  |  | 0.014 | 0.34 | 14.74 |
